# Nature more than nurture affects the growth rate of mussels

**DOI:** 10.1038/s41598-020-60312-y

**Published:** 2020-02-26

**Authors:** D. Prieto, D. Tamayo, I. Urrutxurtu, E. Navarro, I. Ibarrola, M. B. Urrutia

**Affiliations:** GIU 17/061, GI 609, Departamento de Genética, Antropología Física y Fisiología Animal, Facultad de Ciencia y Tecnología, Universidad del País Vasco/Euskal Herriko Unibertsitatea, UPV/EHU, Apartado 644, 48080 Bilbao, Spain

**Keywords:** Physiology, Animal physiology

## Abstract

We tested the hypothesis that environmental trophic conditions prominent during the growing period (nurture conditions) can modify the differing physiological profiles between fast (F)- and slow (S)-growing juveniles of the mussel *Mytilus galloprovincialis*. Approximately 200 individuals were fed a high organic content diet dosed below the pseudofaeces threshold (BP), whereas another 200 were fed a low organic content diet dosed above the pseudofaeces threshold (AP), forcing them to maintain a continuous production of pseudofaeces. After 3 months, F and S individuals in each rearing condition were selected and used in feeding experiments. We measured the physiological parameters of the energy balance of selected F and S mussels fed on 4 different diets and tested the effects of the rearing condition (BP vs AP) and growth condition (F vs S) upon the physiological variables. Irrespective of the rearing condition, F-mussels attained higher values of scope for growth with the four experimental diets due to their capacity to display higher clearance rates and preingestive selection efficiencies. F-individuals also had higher gill-surface areas than S individuals. We discussed the role of the gills in determining inter-individual growth rate differences in the mussel.

## Introduction

An outstanding characteristic of the growth process in bivalve populations is that the growth rate varies enormously between individuals even when they are reared under identical environmental conditions^1–8^ indicating an intense influence of the genetic background on the growth potential of these organisms. Currently, there is no doubt that growth is a genetically determined trait in bivalves^5,9^ that mediates high heritability^10–14^. However, there is still scarce knowledge about the genetic factors contributing to inter-individual growth-rate differences. Growth is a complex phenotypic trait likely controlled by several genetic factors. Not surprisingly, gene expression rates obtained from transcriptomic analysis have shown that fast (F) and slow (S) growing specimens differ in the expression of large amounts (up to thousands) of genes involved in many biological functions^14–17^.

The main physiological components in the processes of growth are summarized in the energy balance equation, where growth is the difference between energy acquisition (absorption) and energy expenditure (metabolic rate). In good correspondence with genetic and transcriptomic results, analysis of the physiological differences between F versus S growing specimens has reported heterogeneous results, revealing that genetic differences causing growth variability affect various physiological components of the energy balance^4–7^^,^^18–20^. To systematize the results and provide a comprehensive frame to analyse the reported differences in the physiological basis of fast growth, Bayne^1^ formulated three non-mutually exclusive physiological models: i) the *acquisition model* (genetic differences affect the capacity of the individuals to acquire and assimilate food), ii) the *allocation model* (genetic differences promote inter-individual differences in the rate of energy allocation for maintenance, growth or reproduction), and iii) the *metabolic efficiency model* (genetic differences affect the metabolic costs of growth).

Suspension feeding in bivalves involves the pumping and filtration of seawater through the gills to capture suspended particles and, under certain conditions of high particle concentration (above the pseudofaeces-production threshold), the preingestive sorting of particles that may be involved in pseudofaeces formation. Eco-physiological studies have demonstrated that bivalves have considerable phenotypic plasticity in modulating feeding rates and selective activity at the gill and labial palps in response to changes in food availability (^21^, for review). The preferential rejection of organically poor particles within the pseudofaeces promotes the organic enrichment of ingested food, thus contributing to increased efficiency of the subsequent processes of digestion and absorption and, hence, the scope for growth (^21^, for review). In an attempt to untangle the complex physiological basis underlying inter-individual differences in the growth rate of bivalves, Tamayo *et al*.^7^ tested the hypothesis that the environmental conditions prevailing during the growing period (nurture), particularly nutritional conditions such as food abundance, could modify the physiological profiles differing between fast and slow growing juveniles of the mussel *M. galloprovincialis*. They showed that with a continuous food supply, mussels that grew at higher rates (fast growers: F) differed from slow growers (S) in their innate capacity to display higher feeding rates, whereas in mussels reared under restrictive feeding conditions, the advantageous innate feature in fast growers (F) was their capacity to reduce standard metabolic rates during starvation periods.

To further analyse how physiologic, genetic and environment interactions determine fast growing in the mussel *M.galloprovincialis*, Prieto *et al*. (^8,17^ and present study) designed a series of long-lasting laboratory experiments where stocks of juvenile specimens were reared and left to size-differentiate (F vs S) under a set of different experimental conditions. In Prieto *et al*.^8^, we reported the results obtained with two groups of mussels that were reared in the laboratory under moderate and severe food-restrictive conditions. The results described in Prieto *et al*.^8^ confirmed those in Tamayo *et al*.^7^ and led us to hypothesize the existence of two fast-growing phenotypes. The specimens genetically well-equipped to acquire and process food that were determined to be fast growers under moderate restrictive feeding conditions were referred to as *fast feeders*. The other phenotype, referred to as *energy savers*, is represented by specimens genetically capable of displaying reduced standard metabolic rates and that were found to be faster growing individuals under severe food-restrictive conditions.

In the present study, we describe the results obtained in a series of experiments that were performed simultaneously with those in Prieto *et al*.^8^, where two groups of mussel spats were reared and left to size-differentiate under conditions of continuous supply of food: one group was fed a low organic content food at a particle concentration above the pseudofaeces threshold (AP), and the other group was fed a high organic content food at a particle concentration below the pseudofaeces production threshold (BP). By rearing the mussels under such different feeding environments, we aimed to evaluate endogenously determined putative differences in the feeding rates and preingestive sorting and pseudofaeces production as potential factors contributing to inter-individual differences in growth potential. We hypothesized that:

1. A better (endogenous) performance at the preingestive level would be one of the key features of the physiological profile of mussels found to be fast growers in the AP condition (F_AP_) compared to mussels found to be slow growers (S_AP_).
2. The physiological basis of fast growth would be different between mussels reared under the nutritional conditions above or below the pseudofaeces production threshold.
3. Differential physiological traits driven by different nurturing conditions would be persistent enough to make mussels reared under both maintenance conditions to respond differentially when exposed to changes in the quality or quantity of the food.

## Results

### Growth rates and selection of fast and slow growers

The growth rates (GR: mm/day) of the mussels reared with both diets, below (BP) and above (AP) pseudofaeces threshold, were estimated by adjusting linear regression models to the variations of the mean values of shell lengths with time (days). The resulting equations were as follows:

$$BP:0.139(\,\pm 0.003)\ast time+\,6.889(\,\pm \,0.088),{\rm{F}}=3,\,078.4,\,p < 0.0001$$

$$AP:\,0.130(\,\pm \,0.002)\ast time+8.524(\,\pm \,0.119),\,{\rm{F}}=4,\,386.3,\,p < 0.0001$$

**Figure 1:**
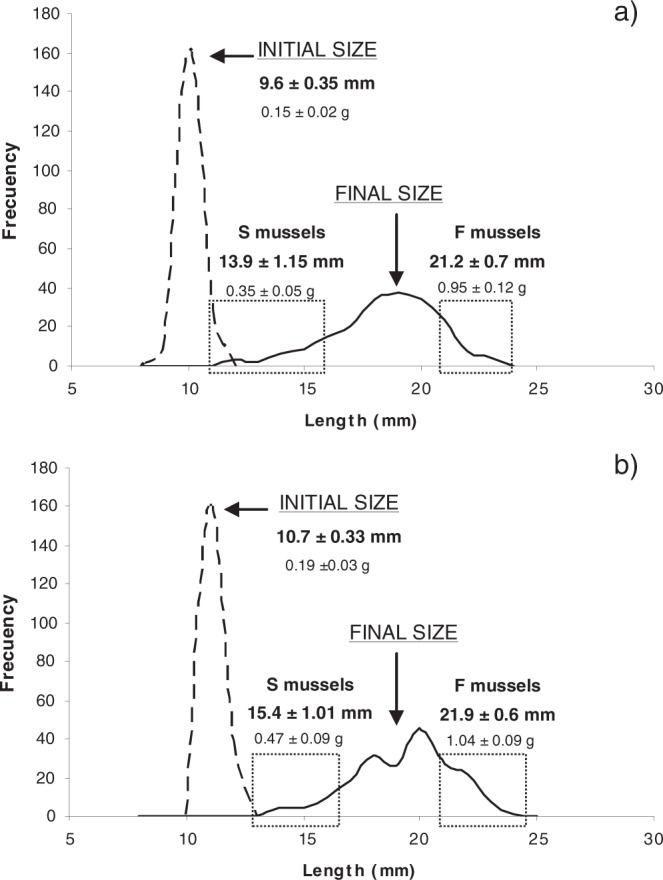
Shell-length distribution of the mussels upon arrival to the laboratory (initial size) and after three months of maintenance (final size) under BP (**a**) or AP (**b**) conditions. The boxes delimit the size range of the selected F and S mussels; their corresponding shell-lengths (mm) and live weights (g) (mean ± SD) are indicated.

Mussels grew an average of 0.13 mm/day under both maintenance conditions. ANCOVA indicated a lack of significant differences in growth rates (slope test: t = 1.77, df=1, 5, p < 0.05; “intercept” test: t = −34.54, df=1, 5, p > 0.05). Inter-individual differences in the growth rates under each rearing condition were so apparent that a rearing period of 3 months was long enough to easily select fast and slow growers from each condition. The live weight of F individuals was 2.5-fold higher than that of S individuals, and the shell length was 45% larger in F individuals than in S individuals (Fig. 1).

### Feeding experiments with selected fast and slow growers

Individuals belonging to the four groups of mussels (F_BP_, S_BP_, F_AP_ and S_AP_) were fed four experimental diets: (i) high-quality, low concentration (H_L_); (ii) high-quality, high concentration (H_H_); (iii) low-quality, low concentration (L_L_); and iv) low-quality, high concentration (L_H_). The organic content (fraction) of the high- and low-quality diets, approximately 0.8 and 0.4, respectively (H_L_ = 0.83 ± 0.06; H_H_ = 0.80 ± 0.03; L_L_ = 0.49 ± 0.07; L_H_ = 0.37 ± 0.10), were similar to those in the *maintenance conditions* (BP and AP). The concentration of particulate organic matter (POM) was approximately 0.4 mg/L for the diets supplied at low concentrations (0.49 ± 0.01 and 0.34 ± 0.03 for H_L_ and L_L_, respectively) and approximately 1.4 mg/L for the diets supplied at high concentrations (1.17 ± 0.12 and 1.68 ± 0.33 for H_H_ and L_H_, respectively).

All physiological parameters recorded for the mussels fed with the four experimental diets are shown in Table 1.

**Table 1:** Physiological parameters (mean ± SD) measured in mussels during feeding experiments: i) high-quality low concentration (H_L_), ii) high-quality high concentration (H_H_), iii) low-quality low concentration (L_L_) and iv) low-quality high concentration (L_H_). Mussel groups: i) fast growers fed below pseudofaeces threshold (F_BP_), ii) slow growers fed below pseudofaeces threshold (S_BP_), iii) fast growers fed above pseudofaeces threshold (F_AP_), iv) slow growers fed above pseudofaeces threshold (S_AP_). Physiological parameters: CR: clearance rate (L/h), SE: selection efficiency (fraction), RP: rejection proportion, OIR: organic ingestion rate (mg/h), AE: absorption efficiency (fraction), AR: absorption rate (mg/h), VO_2R_: routine oxygen consumption (mL/h), VO_2S_: standard oxygen consumption (mL/h) and SFG: scope for growth (J/h). Letters indicate lack of significant difference between growth groups per parameter according to the corresponding post hoc test. N = 6 each experimental group when fed each H_L_, H_H_ and L_L_ diets. N = 8 when fed L_H_ diet.

H_L_diet	F_BP_	S_BP_	F_AP_	S_AP_
	Mean ± SD	Mean ± SD	Mean ± SD	Mean ± SD
CR (L/h)	0.66 ± 0.10^a^	0.30 ± 0.14^b^	0.48 ± 0.17^a.b^	0.40 ± 0.14^b^
OIR (mg/h)	0.32 ± 0.05	0.15 ± 0.07	0.24 ± 0.08	0.19 ± 0.07
AE (fraction)	0.71 ± 0.07^a^	0.78 ± 0.08^a.b^	0.72 ± 0.03^a.b^	0.82 ± 0.06^b^
AR (mg/h)	0.23 ± 0.05^a^	0.12 ± 0.05^b^	0.17 ± 0.06^a.b^	0.16 ± 0.06^a.b^
VO_2R_ (mL/h)	0.065 ± 0.019^a^	0.053 ± 0.014^a^	0.060 ± 0.007^a^	0.078 ± 0.029^a^
VO_2S_ (mL/h)	0.037 ± 0.008^a^	0.043 ± 0.014^a^	0.035 ± 0.016^a^	0.029 ± 0.014^a^
SFG (J/h)	3.03 ± 0.86^a^	1.11 ± 1.18^b^	2.01 ± 1.17^a.b^	1.33 ± 1.06^a.b^
**H**_**H**_ **diet**				
CR (L/h)	0.39 ± 0.08^a^	0.22 ± 0.05^b^	0.39 ± 0.09^a^	0.20 ± 0.09^b^
OIR (mg/h)	0.47 ± 0.11	0.27 ± 0.14	0.48 ± 0.11	0.24 ± 0.11
AE (fraction)	0.55 ± 0.08^a^	0.68 ± 0.04^b^	0.62 ± 0.03^a.b^	0.67 ± 0.08^b^
AR (mg/h)	0.26 ± 0.08^a^	0.18 ± 0.08^a^	0.30 ± 0.08^a^	0.16 ± 0.08^a^
VO_2R_ (mL/h)	0.075 ± 0.018^a^	0.067 ± 0.017^a^	0.068 ± 0.023^a^	0.043 ± 0.03^a^
VO_2S_ (mL/h)	0.045 ± 0.011^a^	0.052 ± 0.007^a^	0.047 ± 0.02^a^	0.032 ± 0.017^a^
SFG (J/h)	3.37 ± 1.25^a^	2.65 ± 1.62^a^	4.20 ± 1.05^a^	2.62 ± 1.68^a^
**L**_**L**_ **diet**				
CR (L/h)	0.85 ± 0.12^a.c^	0.46 ± 0.14^b^	1.05 ± 0.28^c^	0.51 ± 0.27^a.b^
OIR (mg/h)	0.29 ± 0.04	0.16 ± 0.05	0.36 ± 0.10	0.17 ± 0.09
AE (fraction)	0.76 ± 0.03^a.b^	0.70 ± 0.08^b^	0.79 ± 0.01^a^	0.76 ± 0.03^a.b^
AR (mg/h)	0.22 ± 0.03^a.c^	0.11 ± 0.04^b^	0.28 ± 0.08^c^	0.13 ± 0.07^a.b^
VO_2R_ (mL/h)	0.067 ± 0.011^a^	0.064 ± 0.017^a^	0.057 ± 0.009^a^	0.052 ± 0.012^a^
VO_2S_ (mL/h)	0.038 ± 0.011^a^	0.034 ± 0.013^a^	0.033 ± 0.008^a^	0.043 ± 0.016^a^
SFG (J/h)	2.76 ± 0.60^a.b^	0.80 ± 0.84^c^	4.13 ± 1.57^a^	1.45 ± 1.31^b.c^
**L**_**H**_ **diet**				
CR (L/h)	0.35 ± 0.09^a^	0.20 ± 0.04^b^	0.29 ± 0.10^a.b^	0.20 ± 0.04^b^
RP(fraction)	0.55 ± 0.12^a^	0.47 ± 0.11^a.b^	0.55 ± 0.06^a^	0.41 ± 0.10^b^
SE(fraction)	0.39 ± 0.06^a.b^	0.35 ± 0.05^b^	0.44 ± 0.06^a^	0.38 ± 0.05^a.b^
OIR (mg/h)	0.35 ± 0.07^a^	0.23 ± 0.06^b^	0.34 ± 0.13^a.b^	0.25 ± 0.04^b^
AE(fraction)	0.68 ± 0.04^a.b^	0.61 ± 0.06^b^	0.72 ± 0.04^a^	0.66 ± 0.05^b^
AR(mg/h)	0.24 ± 0.06^a^	0.14 ± 0.03^b^	0.25 ± 0.10^a.b^	0.16 ± 0.03^b^
VO_2R_ (mL/h)	0.072 ± 0.013^a^	0.062 ± 0.018^a^	0.052 ± 0.016^a^	0.058 ± 0.022^a^
VO_2S_ (mL/h)	0.043 ± 0.009^a^	0.035 ± 0.012^a^	0.031 ± 0.010^a^	0.041 ± 0.009^a^
SFG (J/h)	3.05 ± 1.23^a^	1.38 ± 0.77^b^	3.55 ± 1.87^a^	1.91 ± 0.72^a.b^

#### Feeding and preingestive processes

**Figure 2:**
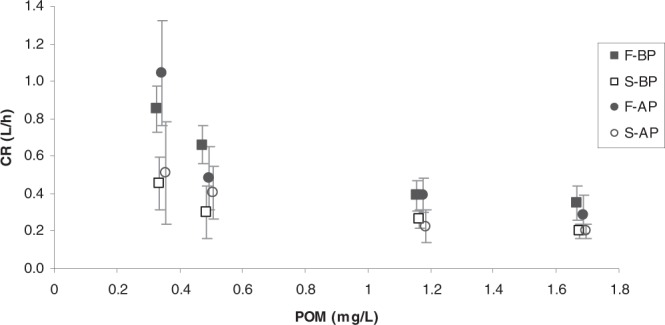
Clearance rate (L/h) (mean ± SD) of F_BP_ , S_BP_ , F_AP_ and S_AP_ mussels as a function of particulate organic matter (POM: mg/L) concentration.

The clearance rates measured for the four mussel groups (F_BP_, S_BP_, F_AP_ and S_AP_) when fed each of the experimental diets were plotted as a function of POM (Fig. 2). Three trends can be highlighted: (a) Clearance rates exponentially decreased with increasing food concentration, irrespective of inter-group differences. (b) CR values corresponding to mussel groups sharing *growth condition* characteristics (fast growers (F_BP_ and F_AP_) versus slow growers (S_BP_ and S_AP_)) were more similar than values corresponding to mussels groups sharing *maintenance condition* characteristics (BP vs AP). (c) Fast-growing mussels (full symbols) displayed systematically higher clearance rates than slow-growing mussels (empty symbols) for the entire range of tested POM concentrations.

The two-factor analysis showed that the *growth condition* factor exerted a highly significant effect on the CR of the four experimental conditions (Table 2), while no significant effect of the *maintenance condition* was observed. A significant effect of the interaction term was recorded only for the H_L_ diet, accounting for the fact that differences in the CRs between fast and slow growing mussels that were reared with diets above pseudofaeces threshold (AP) were very small and not significant.

**Table 2:** P values from two-way factor ANOVA testing for significant effects of *growth condition* (F or S) and *maintenance condition* (BP or AP) on physiological parameters of mussels fed the four experimental diets (H_L_, H_H_, L_L_ and L_H_).

	H_L_	H_H_	L_L_	L_H_
**CR**				
*Maintenance condition*	0.511	0.696	0.170	0.322
*Growth condition*	**0.001**	**0.002**	**<0.001**	**<0.001**
*Interaction*	**0.026**	0.623	0.430	0.270
**RP**				
*Maintenance condition*	n.d.	n.d.	n.d.	0.380
*Growth condition*				**0.006**
*Interaction*				0.375
**SE**				
*Maintenance condition*	n.d.	n.d.	n.d.	**0.037**
*Growth condition*				**0.026**
*Interaction*				0.588
**OIR**				
*Maintenance condition*	0.508	0.701	0.168	0.898
*Growth condition*	**0.001**	**0.002**	**<0.001**	**0.002**
*Interaction*	**0.026**	0.624	0.430	0.543
AE				
*Maintenance condition*	0.313	0.269	**0.024**	**0.015**
*Growth condition*	**0.003**	**0.002**	**0.027**	**0.001**
*Interaction*	0.648	0.129	0.543	0.784
AR				
*Maintenance condition*	0.763	0.583	0.095	0.484
*Growth condition*	**0.013**	**0.010**	**<0.001**	**<0.001**
*Interaction*	**0.035**	0.595	0.402	0.632
VO_2R_				
*Maintenance condition*	0.190	0.111	**0.044**	0.079
*Growth condition*	0.728	0.083	0.445	0.739
*Interaction*	0.065	0.379	0.789	0.239
VO_2S_				
*Maintenance condition*	0.158	0.147	0.623	0.333
*Growth condition*	0.939	0.439	0.578	0.760
*Interaction*	0.273	0.080	0.168	**0.024**
SFG				
*Maintenance condition*	0.387	0.502	**0.042**	0.269
*Growth condition*	**0.009**	0.063	**<0.001**	**0.001**
*Interaction*	0.185	0.472	0.448	0.970

Mussels from the four groups were found not only to have reduced clearance rates but also to produce pseudofaeces when fed the experimental diet L_H_ as a way to limit the ingestion rate. Values corresponding to the proportion of filtered matter that was rejected and the selection efficiency of the pseudofaeces production process recorded for each mussel group are shown in Table 1. Fast growers showed significantly higher rejection rates both in total (in good correspondence with their higher filtration rates) and relative terms: the rejected fraction accounted for 55% and 44% of the filtration rate in F and S mussels, respectively. A significant effect of the *growth condition* factor was confirmed by the results of two-factor ANOVA as shown in Table 2. Post hoc comparison showed that there were significant differences in the rejected proportions between the F_AP_ and S_AP_ mussel groups but not between the F_BP_ and S_BP_ groups.

Regarding the capacity to selectively reject inorganic matter and preferentially ingest organic material, although differences in the indexes reported for each mussel group were small, both the *growth condition* and *maintenance condition* factors (Table 2) were found to exert a significant effect on the selection efficiency (SE): F individuals were better at selecting organic matter than S individuals, and mussels reared in AP conditions were more efficient than their BP counterparts.

**Figure 3:**
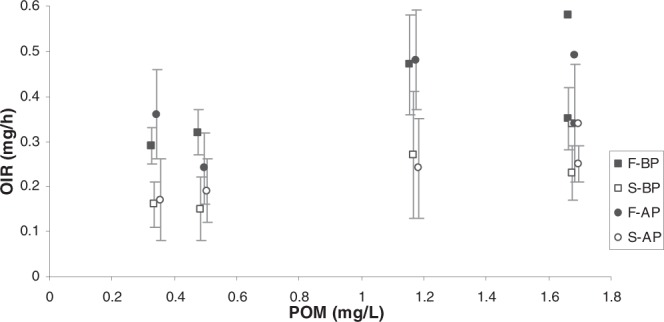
Organic ingestion rate (mg/h) (mean ± SD) of F_BP_ , S_BP_ , F_AP_ and S_AP_ mussels as a function of particulate organic matter (POM: mg/L) concentration. In the POM value representing the L_H_ diet, the organic filtration rates (mg/h) of each mussel group were added (without SD, for clarity).

Irrespective of maintenance condition, and in correspondence with the recorded differences in CR for the H_L_, H_H_ and L_L_ diets, higher rates of organic matter filtration (OFR) and ingestion (OIR) were recorded for F mussels than for S mussels; in the case of the L_H_ diet, the production of pseudofaeces resulted in reduced total and organic ingestion rates in comparison with the L_L_ diet (see Fig. 3), but as a result of the higher selection efficiencies and greater proportion of rejection in F mussels, a significant difference in organic ingestion rate between F and S mussels remained (Fig. 3, Table 1).

#### Digestion and absorption processes

**Figure 4:**
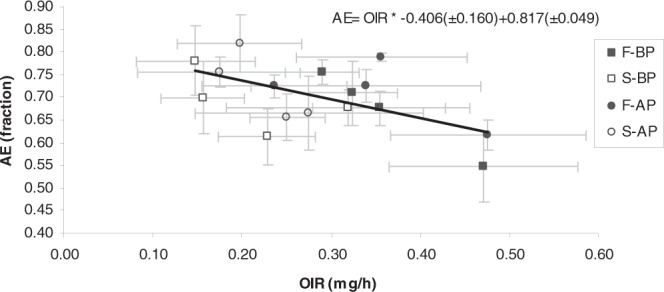
Absorption efficiency (mean ± SD) of the four mussel groups (F_BP_ , S_BP_ , F_AP_ and S_AP_) as a function of organic ingestion rate (mg/h) (mean ± SD).

Figure 4 shows the mean values of the absorption efficiency of food measured for each of the four mussel groups (F_BP_, S_BP_, F_AP_ and S_AP_) when fed each of the four experimental diets as a function of the recorded ingestion rates of organic matter. A trade-off between both parameters was observed: as a rule, mussels with higher organic ingestion rates were found to absorb organic matter from the food with a lower efficiency than mussels ingesting organic particles at lower rates. A linear regression analysis revealed the existence of a significant (p = 0.024) negative correlation between these two parameters.

When addressing differences found between mussel groups fed the different experimental diets, some interesting results were revealed: after testing the effect of the *growth condition* (F vs S) and *maintenance condition* (BP vs AP) factors on the absorption efficiency (see the two-way ANOVA results in Table 2), *growth condition* was found to exert a significant effect on AE for the four feeding conditions. The interpretation of this result is far from clear, however, because while higher values of absorption efficiency were reported for slow-growing mussels fed the high-quality diets than for their fast-growing counterparts, the opposite was found for mussels fed the low-quality diets (Table 1). Therefore, the general rule mentioned above (Fig. 4) was applicable to inter-group differences observed between mussels fed high quality diets but not for inter-group differences observed between mussels fed low quality diets, where fast-feeding mussels were capable of absorbing organic matter more efficiently than slow-feeding mussels.

In addition to the *growth condition* factor, the *maintenance condition* factor was also found to significantly affect AE in mussels fed experimental diets of low organic content (L_L_ and L_H_ diets). AP mussels absorbed ingested organic matter with an efficiency that was slightly but significantly higher than the efficiency reported for BP mussels.

**Figure 5:**
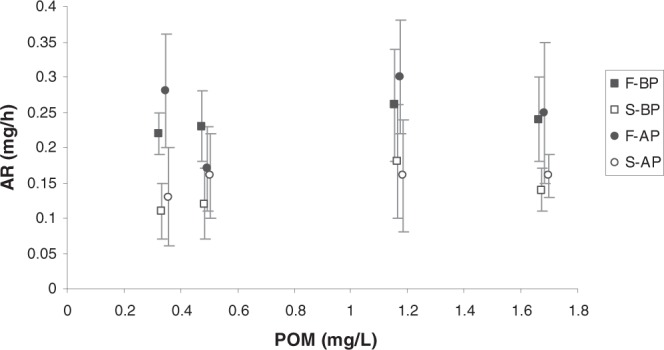
Absorption rate (mg/h) (mean ± SD) of the four mussel groups (F_BP_ , S_BP_ , F_AP_ and S_AP_) as a function of particulate organic matter (POM: mg/L) concentration.

The resulting AR is plotted in Fig. 5 as a function of particulate organic matter (POM). Irrespective of the maintenance condition, fast-growing mussels (full symbols) attained higher rates of food absorption than slow-growing mussels (empty symbols) over the whole range of food concentrations. Significant effects of *growth condition* and *maintenance condition* on absorption rate were tested by two-factor ANOVA as shown in Table 2. The pattern resembles that of CR: i) *growth condition* exerted a significant effect on AR for the four experimental diets, ii) no significant effect of the *maintenance condition* was observed, and iii) the interaction term was significant only for mussels fed the H_L_ diet; as before, this accounted for the fact that almost identical absorption rates were recorded for F and S mussels reared under AP conditions.

#### Routine and standard metabolic rates

**Figure 6:**
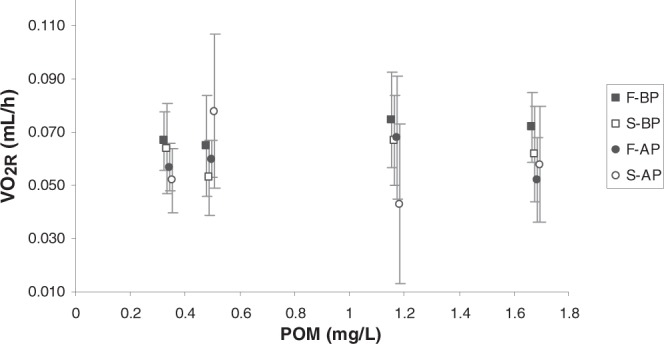
Routine oxygen consumption (mL/h) (mean ± SD) of the four mussel groups (F_BP_ , S_BP_ , F_AP_ and S_AP_) as a function of particulate organic matter (POM: mg/L) concentration.

Mean values of routine oxygen consumption (VO_2R_) obtained for the four mussel groups when fed each one of the experimental diets are plotted as a function of POM in Fig. 6. No clear trend was observed from these data, nor was it observed when analysing standard oxygen consumption (VO_2S_) measured for each of the four groups of mussels after seven days of fasting (see values in Table 1).

To analyse the potential effect of the *growth condition* and *maintenance condition* factors on the routine and standard oxygen consumptions, two-factor ANOVA was performed on the data from mussels fed with each experimental diet (Table 2). The *growth condition* factor did not exert any significant effect on standard or routine oxygen consumption in any of the experimental diets. The *maintenance condition* factor exerted a slightly significant effect (p = 0.044) only on the VO_2R_ of mussels fed the L_L_ diet. This accounted for the fact that mussels reared with the AP diet displayed lower routine oxygen consumption than those mussels reared with the BP diet.

#### Energy balance

**Figure 7:**
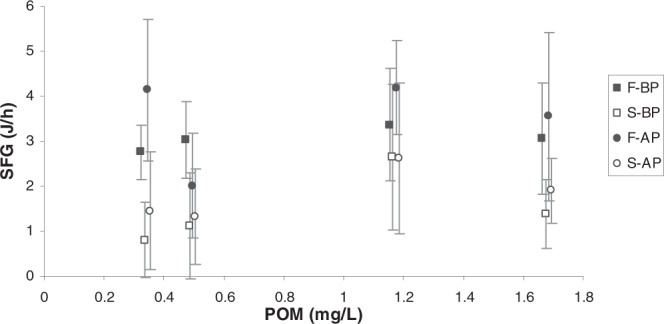
Scope for growth (J/h) (mean ± SD) of the four mussel groups (F_BP_ , S_BP_ , F_AP_ and S_AP_) as a function of particulate organic matter (POM: mg/L) concentration.

The SFG of mussels from the four experimental groups (F_BP_, S_BP_, F_AP_ and S_AP_) fed the four experimental diets are plotted as a function of POM in Fig. 7. Since the VO_2R_ values remained relatively constant (Fig. 6), the differences in SFG resemble those observed in AR (Fig. 5). The figure shows that a) Mussels were able to keep relatively constant values of SFG through the experimental conditions, especially those that were determined to be fast growers; b) SFGs corresponding to mussel groups sharing *growth condition* characteristics (F_BP_ and F_AP_ vs S_BP_ and S_AP_) were more similar than values corresponding to mussels groups sharing *maintenance condition* characteristics (BP vs AP); and c) Fast-growing mussels (full symbols) attained higher SFG values than slow-growing mussels (empty symbols) for the whole range of POM concentrations.

Two-factor analysis of variance (Table 2) showed that *growth condition* exerted a significant effect on the SFG of mussels fed all of the experimental diets except the H_H_ diet (p = 0.063, which was close to statistical significance), while *maintenance condition* exerted a slightly significant effect (p = 0.042) only on mussels fed the L_L_ diet.

### Gill-surface area of selected F and S mussels

**Figure 8:**
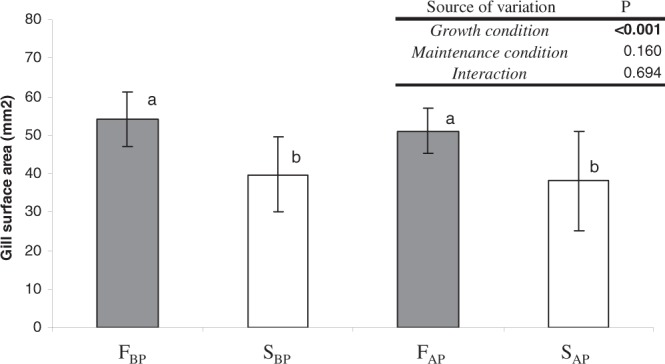
Gill-surface area (mean ± SD) of F_BP_ , S_BP_ , F_AP_ and S_AP_ mussels. Non-significant differences are marked with the same letter. Upper-right: p values of two-way ANOVA testing significant effects of growth condition (F or S) and maintenance condition (BP or AP).

Mean values ( ± SD) of the gill-surface area and the statistical comparison between groups is shown in Fig. 8. Irrespective of the *maintenance condition*, the gill-surface area of F mussels was significantly higher than that of S mussels. Two-way ANOVA performed to test the effect of *growth condition* and *maintenance condition* on the gill-surface area (mm^2^) of the mussels (Fig. 8) confirmed that only the *growth condition* factor exerted a significant effect: F mussels had notably higher gill areas than S mussels.

## Discussion

The physiological components that determine growth rate differences between individuals living under identical environmental conditions may be modulated by differences in i) food acquisition and assimilation, ii) the allocation of energy for maintenance, growth or reproduction, iii) the metabolic costs of growth^1^, or by combinations of some or all of these differences. The scope for growth provides a useful tool for integrating basic physiological processes as a balance of energy that results in a good proxy of the energy available for growth in bivalves (see^22^ for review). In this study, the selection of fast (F)- and slow (S)- growing mussels in the two maintenance conditions, one above (AP) and the other below the pseudofaeces threshold (BP), was based on shell size and live weight measurements of the individual mussels and thus on experimentally measured growth rates. Once fast and slow growers were selected, the potential effects of variables such as the *growth condition* (F or S growers) or the *maintenance condition* (AP or BP) on the physiological parameters determining the energy balance and on the balance itself (SFG) were tested.

Contrary to our expectations as formulated in the hypothesis, the tested nurturing conditions did not have any effect on the differential innate physiological capacities underlying growth capacity, since irrespective of the past feeding history (*maintenance condition*), mussels selected as fast or slow growers under the two maintenance conditions shared common physiological features. The *maintenance condition* factor only exerted a significant effect on some relative indexes, such as AE and SE (in the L_H_ diet). Regarding SE, both *maintenance condition* and *growth condition* factors exerted significant effects, indicating that as a general rule, F mussels selected organic matter for ingestion more efficiently than S mussels and that overall, mussels “trained” in pseudofaeces production were more efficient at rejecting inorganic matter than their counterparts, which had never before produced pseudofaeces. In a companion experiment^8^, using the same rearing and experimental conditions, but with mussels submitted to a tidal emersion regime for of 8 h each day, we found significant differences in SE between F and S mussels. Thus, we tend to give credence to the idea that the *growth condition* exerts a significant effect upon preingestive selection efficiency. The most remarkable feature in the differences in SE between F and S was that such differences were not restricted to mussels reared under conditions that compelled the production of pseudofaeces (AP mussels) but were also present in mussels grown under conditions in which pseudofaeces were never produced (BP mussels). Thus, it seems that a higher ability for preingestive selection is an inherent feature of fast-growing mussels.

Concerning the absorption efficiency, the *maintenance condition* appeared to be a significantly affecting factor when the four mussel groups were fed the low organic content diets (L_L_ and L_H_). In bivalves, AE is positively dependent on the quality of the ingested food. Thus, the slightly higher efficiency of the pallial organs of the AP mussels compared to the BP mussels could have improved the absorptive capacity of these individuals, although we cannot discount that some kind of additional digestive adaptation had occurred during the rearing period.

No effects were observed on the routine oxygen consumption or on the standard oxygen consumption. The absence of significant effects on the VO_2S_ is understandable since after one week of starving, only basic processes of cell maintenance would continue, and those could be similar irrespective of the rearing conditions or even of the growing rate if differential growing rates were not based on differences in the energy allocation processes. Routine metabolic rate has been shown to be a rising function of absorption rate in bivalves^1,23–25^. Strikingly, our results did not show any significantly higher metabolic rate with increasing AR. Similar metabolic rates were measured in F and S mussels despite AR being two-fold higher in F mussels. This result suggests that, in addition to a higher capacity to acquire and process food, F mussels seem to have a higher metabolic efficiency and/or lower costs of growth, which also constitutes a feature promoting faster growth.

Thus, the obtained results do not support the first nor the second hypothesis formulated in the introduction. As we expected, F_AP_ mussels displayed significantly higher preingestive selection efficiencies than S_AP_ mussels; however, the same difference was also observed between F_BP_ and S_BP_ mussels, so it must be concluded that a better capacity to cope with high seston loads is an inherent trait of fast growing mussels and not the result of an adaptation to a turbid environment.

Contrary to the expectations outlined in the second hypothesis, the physiological basis for differential growth was found to be exactly the same between mussels reared above or below the pseudofaeces threshold. Inter-individual growth potential differences are caused by the existence of endogenously determined differences in the feeding rate and the efficiency of the preingestive processes for particle selection. Thus, the obtained results strongly support an increased energy acquisition model (as defined by Bayne^1^) for explaining differences in the growing rate between F and S mussels. Irrespective of the rearing condition, fast growing mussels showed significantly higher CRs (two-three times higher in some experimental diets) than slow growers in all the experimental diets, promoting significantly higher organic ingestion rates. This was also observed for the L_H_ diet, where F mussels rejected a larger proportion of filtered matter and better selected food items, conferring them significantly higher OIRs in comparison with the slow-growing individuals. Although higher OIRs recorded in F mussels sometimes resulted in slightly lower AEs that limited absorptive capacity, the effect was not strong enough to counteract the benefits derived from ingesting more food, since significantly higher absorption rates were measured systematically for fast growers. Thus, SFG differences recorded between F and S mussels corresponded to enhanced feeding rates and higher digestive performance of F mussels (acquisition model as defined by Bayne^1^), which jointly reduced metabolic costs per unit of assimilated food compared to S mussels (metabolic efficiency model as defined by Bayne^1^). These results are consistent with those previously reported not only for mussels but also for oysters and clams^2–6,8,20,26–29^.

The third hypothesis stated in the introduction addresses the possibility that mussels reared under different maintenance conditions would respond differentially when exposed to changes in food quality and quantity. Bivalves have a considerable capacity to adjust feeding rates and digestive parameters to cope with short- (hours, days) and medium-term (weeks) variations in trophic conditions^18,21,23,24,30–34^. The regulation of ingestion rate by modifying CR and/or by producing pseudofaeces in addition to a preferential ingestion of organic matter are the most remarkable adaptations to such variations. In the present experiment, the variations in the physiological parameters among the experimental diets fit the expected responses in terms of physiological modification to variation in food characteristics: an increased particle concentration of high-quality food (from H_L_ to H_H_) promoted a general reduction of the clearance rate that led to the cancellation of most of the significant differences in the SFG among the groups. However, a differential response between F and S mussels has been found: fast growers were able to modify their CR more markedly than S individuals (see the increase at low food concentrations as an example in Fig. 2), and consequently, they were able to better compensate for food variations, so that their SFG remained less variable (see Fig. 7).

The measurements performed in the present study showed that fast growing mussels from both maintenance conditions possessed significantly larger gill-surface areas than their S counterparts (Fig. 8). This result is entirely consistent with the physiological data showing that fast growth is based, irrespective of the maintenance condition, on the capacity of F specimens to display higher clearance rates and selection efficiencies than S specimens. An analysis of the transcriptome of the gills of these experimental mussels^17^ has shown the existence of broad differences in the genetic expression of the gill tissue between fast and slow growing specimens that likely explain the corresponding physiological and morphometric differences: the gills of S mussels suffer a greater stress either because they have a greater prevalence of pathogens/diseases or because they have a higher susceptibility to pathogens that force them to devote more metabolic energy to the maintenance of immune and defence processes to ensure survival at the expense of growth rate.

The difference in the immune responses found between fast and slow growing specimens revealed by a gill-transcriptomic analysis in Prieto *et al*.^17^ constitutes a relevant endogenous factor causing inter-individual differences in the growth potential of the mussels from the present experiments and might also explain those observed in Prieto *et al*.^8^. A putative, higher metabolic requirement linked to a less-efficient immune system could be a relevant physiological factor limiting the scope for growth of S individuals and could ultimately promote the size-differentiation between F and S specimens. In conditions of continuous food supply (of either high or low quality), the differential energetic investments in supporting defence mechanisms would lead to the existence of significant differences in the energetic scopes for feeding and growth between F and S specimens. Such a differential constraint would lead to the observed differences between fast and slow growers in the physiological parameters characterizing food acquisition (*fast feeders*), such as clearance-ingestion-absorption rates and preingestive selection efficiency that were observed in Prieto *et al*.^8^ and in the present experiment. At the same time, the higher metabolic demands of the immune system would limit the capacity of slow-growing individuals to reduce the standard metabolic rate during the periods of starvation. Such a differential trait could explain the size differentiation between F and S specimens based on the existence of significant differences in standard metabolic rate (*energy savers*) in mussels subject to severe feeding restrictions, such as those observed in Prieto *et al*.^8^ and Tamayo *et al*.^7^. Moreover, such differences in energy investment might also determine the capacity of mussels to spend energy resources on gill development. In turn, since the gill-surface area seems to limit acquisition rate, such differences could be the main endogenous differences underlying the inter-individual growth rate variability in bivalves.

## Materials and Methods

### Experimental design

Approximately 400 juveniles of 10 mm shell length of the mussel *M. galloprovincialis* were collected from the rocky shores of Antzoras (Bizcay, North Spain, 43 °24′29.1″N; 2 °40′51.0″W) in February 2014. At the laboratory, mussels were separated into two groups of about 200 individuals. Both groups were maintained in two sea water-containing tanks (volume of each tank: 50 L) at constant temperature (16 °C) and water salinity (33 PSU). Tanks were cleaned daily, and mussels were pulled apart one from each other to avoid inter-individual competition for food. Mussels were continuously fed with mixtures of the algae *Isochrysis galbana* (T-iso), lyophilized *Phaeodactilum tricornutum* and freshly collected and sieved particles of natural sediment. One group was fed a high organic content diet (80%) at a concentration below the pseudofaeces threshold (BP diet: 1–1.5 mm^3^/L), whereas the second group was fed a low organic content diet (30%) at a concentration above the pseudofaeces threshold (AP diet: 3–3.5 mm^3^/L). The concentration of particulate organic matter (POM) in the BP and AP diet tanks were ≈ 0.8 and 1.6 mg/L respectively. Diets were continuously pumped into the tanks by peristaltic pumps from concentrated stocks. The concentrations in the tanks were maintained stable by checking frequently with a Coulter Multisizer 3. The shell lengths and live weights of individual mussels were measured once every two weeks using 0.05 mm accuracy calipers and a 0.01 mg accuracy balance. Mussels were maintained under these conditions until large inter-individual size differences were found (3 months). After this period, the 26 largest and smallest individuals from each group were selected and were denoted, respectively, as fast (F) and slow (S) growers. Thus, four experimental mussel groups resulted from the combination of the *maintenance condition* (BP and AP) and *growth condition* (F or S):

1. Fast growers selected below the pseudofaeces threshold (F_BP_);
2. Slow growers selected below the pseudofaeces threshold (S_BP_);
3. Fast growers selected above the pseudofaeces threshold (F_AP_); and
4. Slow growers selected above the pseudofaeces threshold (S_AP_).

To analyse possible differences in the physiological performance of the fast- and slow-growing mussels reared under the different maintenance conditions, 6 to 8 individuals belonging to these 4 groups were used in a series of feeding experiments, and the physiological parameters determining the energy balance were measured. Four experimental diets were used in the feeding experiment, resulting from the combination of 2 food qualities x 2 food concentrations: i) high-quality, low concentration (H_L_); ii) high-quality, high concentration (H_H_); iii) low-quality, low concentration (L_L_); and iv) low-quality, high concentration (L_H_). The characteristics of the H_L_ and L_H_ experimental diets were similar to the BP and AP diets given to the mussels during maintenance.

### Feeding experiments performed with the selected fast (F)- and slow (S)-growing juveniles

#### Characteristics of experimental diets

Experimental diets were made up by mixing cells of the microalgae *Isochrysis galbana* (T-iso) and silt particles. Gravimetric characteristics of each diet were determined every day during energetic physiology experiments. Water samples from the experimental tanks were filtered onto ash, pre-weighed GF/C glass-fibber filters and subsequently processed to determine total particle matter (TPM: mg/L), particulate inorganic matter (PIM: mg/L) and particulate organic matter (POM: mg/L) concentrations. Retained salts were rinsed out with a solution of ammonium formate (0.9%). TPM and PIM were estimated as the dry and ash weight increases of the filters, respectively. POM was calculated as the difference between TPM and PIM. Organic content (f) was estimated as POM/TPM.

#### Determination of the physiological parameters

Mussels were acclimated for one week to the experimental diets before measuring the physiological rates. Six individuals (n = 6) from each experimental group (F_BP_, S_BP_, F_AP_, S_AP)_ were used to test H_L_, H_H_ and L_L_ diets, whereas 7–8 individuals from each group were used for testing the L_H_ diet, where differences in the production of pseudofeces were expected. The physiological rates were determined as described below:

Energy acquisition: For clearance rate determinations, mussels were placed in plastic cylindrical chambers (500 mL) that formed part of the top of a filtering device consisting of an inverted conical flask serving as a filtration chamber and suited for the simultaneous collection of faeces with minimal disturbance of mussels (see Tamayo *et al*.^29^ for further description). Water from a feeding tank containing the diet was pumped through the feeding chambers by means of a pump. The flow rate in each individual chamber was manually regulated and adjusted to produce flow rates appropriate to achieve 15–30% reduction in particle concentration inside the chambers. The particle suspension in the feeding tank was maintained at the correct concentration (mm^3^/L) by the addition of the appropriate amount of particles delivered from a concentrated stock of the diet. A control chamber (without animal) served to correct for particle sedimentation.

*Clearance rate* (CR: L/h) was measured according to Hildreth and Crisp^35^ as follows:

$${\rm{CR}}={\rm{F}}\ast (({\rm{Ci}}-{{\rm{C}}}_{0})/{\rm{Ci}})$$

where F is the flow rate (L/h), Ci is the particle concentration in the control outflow and C_0_ is the particle concentration in the outflow of the experimental chamber. Particle concentration was determined with a Coulter Z1 counter. Samples of water in the outflow of individual and control chambers were taken every hour during a period of 11–12 h. Thus, the CR value of each individual was calculated by averaging the 11 to 12 measurements taken throughout day.

*Filtration rates* of the total (FR: mg/h) and particulate organic matter (OFR: mg/h) were calculated as the product of CR*TPM and CR*POM, respectively. When mussels were fed below the pseudofaeces threshold (H_L_, H_H_ and L_L_ diets), both FR and OFR were equivalent to the ingestion rates of total (IR: mg/h) and organic matter (OIR: mg/h), respectively.

*Absorption efficiency* (AE: decimal units) was determined according to Conover^36^, comparing the organic content of the experimental tank water (f) and the organic content of the faeces collected from each of the mussels (h):

$${\rm{AE}}=({\rm{f}}-{\rm{h}})/(1-{\rm{f}})\ast {\rm{h}}$$

The resulting *absorption rate* (AR: mg/h) was computed as the product of OIR and AE.

The mussels fed the experimental diet L_H_ produced pseudofaeces, and accordingly, the absorption rate was calculated as follows: the pseudofaeces produced while measuring the clearance rate were collected to calculate the rejection rate of total and organic particulate matter (RR and ORR, respectively: mg/h). The proportion of filtered matter that was rejected in pseudofaeces (RP) was computed as RR/FR. The ingestion rate and organic ingestion rate were then computed as the difference between filtration and rejection rates (IR = FR-RR and OIR = OFR-ORR, respectively). The faeces produced by each individual during the period of CR measurement were collected to calculate the egestion rate of total and organic matter (ER and OER, respectively: mg/h). The resulting absorption rate (AR) was computed as OIR-OER, and the absorption efficiency was calculated as AR/OIR.

The preingestive selection efficiency (SE: fraction) was determined according to Kiørboe and Møhlenberg^37^ as SE = 1 - (p /f), where p is the organic content of the pseudofaeces and f is the organic content of the food.

Metabolic expenditures: After the determination of food acquisition rates, mussels were introduced into individual chambers (150 mL) sealed with LDO oxygen probes connected to oximeters (HATCH HQ 40d) for the determination of routine oxygen consumption (VO_2R_: mL O_2_/h). The rates of VO_2_ were derived from the decrease in the oxygen concentration of the water over time. Oxygen concentration was measured every 5–10 minutes until the values had decreased by 20–30% of the baseline. A control chamber was used to check the stability of the oxygen concentration. Once the routine oxygen consumption was measured, mussels were starved for seven days, and the rates of oxygen consumption were measured again to determine the standard oxygen consumption (VO_2S_: mL O_2_/h).

The *routine metabolic rate* (RMR: J/h) and *standard metabolic rate* (SMR: J/h) were estimated from the routine and standard oxygen consumption, respectively using an oxycaloric coefficient of 20.08 J/mL O_2_^38^.

Energy balance: *Scope for growth* (SFG: J/h) was estimated as the difference between the absorption rate (AR) and the routine metabolic rate (RMR). The AR (mg/h) was transformed into energy units (J/h) by using the energy content for *Isochrysis galbana* of 18.75 J/mg^39^.

#### Size Standardization

Physiological determinations were expressed in terms of live weight. Clearance rates and oxygen consumptions were standardized to a common live weight of 1 gr. according to the following expression^22^:

$${{\rm{Y}}}_{{\rm{STD}}}={(1/{{\rm{W}}}_{\mathrm{EXP}})}^{{\rm{b}}}\ast {{\rm{Y}}}_{\mathrm{EXP}},$$

where Y_STD_ and Y_EXP_ represent the standard and experimental physiological rates, respectively. W_EXP_ is the experimental weight of the mussel, and b is the power value that scales physiological rates to body weight. The allometric values (b) used for clearance rate and oxygen consumption were 0.58^40^ and 0.724^41^, respectively.

#### Gill-surface area (GA: mm^2^)

Once the physiological parameters were measured, the animals were carefully dissected by cutting the abductor muscles. The mussels were placed on graph paper to set the scale, and a photograph of the internal tissues of each mussel was taken with a digital camera. The gill surface area was estimated from the photo with the ImageJ program. Displayed data correspond to one side of a demibranch. Gill areas were standardized for an equivalent 1 gr. live-weight mussel according to the expression:

$${{\rm{GA}}}_{{\rm{STD}}}={(1/{{\rm{W}}}_{\mathrm{EXP}})}^{{\rm{b}}}\ast {{\rm{GA}}}_{\mathrm{EXP}},$$

where GA_STD_ and GA_EXP_ represent the standardized and experimental gill areas, respectively, and W_EXP_ is the experimental live weight of the mussel. The power function that scales gill area to live weight is 0.66^42–44^.

Gills were dissected, then immersed in RNA and used for gill-transcriptomic analysis^17^.

#### Statistical analysis

Normality and homogeneity of variances were tested using Shapiro-Wilk and Levene tests, respectively, prior to analysis of the data. Significant differences in growth rates between mussels grown with BP and AP diets were tested by comparing the slope (b) and intercepts (a) using analysis of covariance (ANCOVA) procedures described in Zar^45^. The significance level of the effect that the *growth condition* factor (this is, the effect of being fast or slow grower), the *maintenance condition* factor (this is, having been reared under BP or AP diet), and their interaction might exert on the physiological parameters measured were tested using two-way ANOVA. Differences between groups were analysed by post hoc tests, Games Howell or Tukey, according to Levenne test results. Statistical analyses were performed using IBM SPSS Statistics 19.

## Supplementary information

Supplementary information

## Data Availability

The datasets generated and analysed during the current study are available in the Marine Data Archive repository, http://mda.vliz.be/directlink.php?fid=VLIZ_00000738_5d44796e30a2a.
