# Supplementary material for: Nature more than nurture affects the growth rate of mussels
Source: Sci Rep. 2020 Feb 26;10:3539. doi: 10.1038/s41598-020-60312-y (PMC7044158; doi:10.1038/s41598-020-60312-y)
Supplement: Supplementary file 1 — Supplementary information [file 41598_2020_60312_MOESM1_ESM.doc]

**Nature more than nurture affects the growth rate of mussels**

D. Prieto^*^, D. Tamayo, I. Urrutxurtu, E. Navarro, I. Ibarrola and M.B. Urrutia

**Supplementary information**

**Figure A.1.** Experimental design.

**Table A1. Two-way factor ANOVA testing significant effects of growth condition (F or S) and maintenance condition (BP or AP) on physiological parameters of mussels when fed the four experimental diets (H_L_, H_H_, L_L_ and L_H_).**

| H_L_ diet |  |  |  |  |  |
| --- | --- | --- | --- | --- | --- |
| **Source of variation** | **DF** | **SS** | **MS** | **F** | **P** |
| CR |  |  |  |  |  |
| *Maintenance condition* | 1 | 0.009 | 0.009 | 0.449 | 0.511 |
| *Growth condition* | 1 | 0.276 | 0.276 | 14,351 | 0.001 |
| *Interaction* | 1 | 0.112 | 0.112 | 5,841 | 0.026 |
| *Residual* | 19 | 0.365 | 0.019 |  |  |
| OIR |  |  |  |  |  |
| *Maintenance condition* | 1 | 0.002 | 0.002 | 0.456 | 0.508 |
| *Growth condition* | 1 | 0.066 | 0.066 | 14445 | 0.001 |
| *Interaction* | 1 | 0.027 | 0.027 | 5,862 | 0.026 |
| *Residual* | 19 | 0.087 | 0.005 |  |  |
| AE |  |  |  |  |  |
| *Maintenance condition* | 1 | 0.004 | 0.004 | 1,069 | 0.313 |
| *Growth condition* | 1 | 0.041 | 0.041 | 11,004 | 0.003 |
| *Interaction* | 1 | 0.001 | 0.001 | 0.215 | 0.648 |
| *Residual* | 20 | 0.074 | 0.004 |  |  |
| AR |  |  |  |  |  |
| *Maintenance condition* | 1 | 0.000 | 0.000 | 0.093 | 0.763 |
| *Growth condition* | 1 | 0.023 | 0.023 | 7489 | 0.013 |
| *Interaction* | 1 | 0.016 | 0.016 | 5149 | 0.035 |
| *Residual* | 19 | 0.059 | 0.003 |  |  |
| VO2R |  |  |  |  |  |
| *Maintenance condition* | 1 | 0.001 | 0.001 | 1841 | 0.190 |
| *Growth condition* | 1 | 0.000 | 0.000 | 0.124 | 0.728 |
| *Interaction* | 1 | 0.001 | 0.001 | 3822 | 0.065 |
| *Residual* | 20 | 0.007 | 0.000 |  |  |
| VO2S |  |  |  |  |  |
| *Maintenance condition* | 1 | 0.000 | 0.000 | 2150 | 0.158 |
| *Growth condition* | 1 | 0.000 | 0.000 | 0.006 | 0.939 |
| *Interaction* | 1 | 0.000 | 0.000 | 1270 | 0.273 |
| *Residual* | 20 | 0.003 | 0.000 |  |  |
| SFG |  |  |  |  |  |
| *Maintenance condition* | 1 | 0.906 | 0.906 | 0.783 | 0.387 |
| *Growth condition* | 1 | 9,666 | 9,666 | 8354 | 0.009 |
| *Interaction* | 1 | 2189 | 2189 | 1892 | 0.185 |
| *Residual* | 19 | 21983 | 1157 |  |  |

| H_H_ diet |  |  |  |  |  |
| --- | --- | --- | --- | --- | --- |
| **Source of variation** | **DF** | **SS** | **MS** | **F** | **P** |
| CR |  |  |  |  |  |
| *Maintenance condition* | 1 | 0.002 | 0.002 | 0.157 | 0.696 |
| *Growth condition* | 1 | 0.128 | 0.128 | 12.790 | 0.002 |
| *Interaction* | 1 | 0.002 | 0.002 | 0.249 | 0.623 |
| *Residual* | 20 | 0.200 | 0.010 |  |  |
| OIR |  |  |  |  |  |
| *Maintenance condition* | 1 | 0.002 | 0.002 | 0.152 | 0.701 |
| *Growth condition* | 1 | 0.187 | 0.187 | 12.780 | 0.002 |
| *Interaction* | 1 | 0.004 | 0.004 | 0.248 | 0.624 |
| *Residual* | 20 | 0.292 | 0.015 |  |  |
| AE |  |  |  |  |  |
| *Maintenance condition* | 1 | 0.005 | 0.005 | 1.290 | 0.269 |
| *Growth condition* | 1 | 0.047 | 0.047 | 12.345 | 0.002 |
| *Interaction* | 1 | 0.010 | 0.010 | 2.512 | 0.129 |
| *Residual* | 20 | 0.076 | 0.004 |  |  |
| AR |  |  |  |  |  |
| *Maintenance condition* | 1 | 0.002 | 0.002 | 0.312 | 0.583 |
| *Growth condition* | 1 | 0.049 | 0.049 | 8.115 | 0.010 |
| *Interaction* | 1 | 0.002 | 0.002 | 0.293 | 0.595 |
| *Residual* | 20 | 0.115 | 0.006 |  |  |
| VO2R |  |  |  |  |  |
| *Maintenance condition* | 1 | 0.001 | 0.001 | 2.775 | 0.111 |
| *Growth condition* | 1 | 0.002 | 0.002 | 3.335 | 0.083 |
| *Interaction* | 1 | 0.000 | 0.000 | 0.810 | 0.379 |
| *Residual* | 20 | 0.010 | 0.001 |  |  |
| VO2S |  |  |  |  |  |
| *Maintenance condition* | 1 | 0.000 | 0.000 | 2.282 | 0.147 |
| *Growth condition* | 1 | 0.000 | 0.000 | 0.624 | 0.439 |
| *Interaction* | 1 | 0.001 | 0.001 | 3.397 | 0.080 |
| *Residual* | 20 | 0.004 | 0.000 |  |  |
| SFG |  |  |  |  |  |
| *Maintenance condition* | 1 | 0.945 | 0.945 | 0.468 | 0.502 |
| *Growth condition* | 1 | 7.802 | 7.802 | 3,863 | 0.063 |
| *Interaction* | 1 | 1.086 | 1.086 | 0.538 | 0.472 |
| *Residual* | 20 | 40.396 | 2.020 |  |  |

| L_L_ diet |  |  |  |  |  |
| --- | --- | --- | --- | --- | --- |
| **Source of variation** | **DF** | **SS** | **MS** | **F** | **P** |
| CR |  |  |  |  |  |
| *Maintenance condition* | 1 | 0.093 | 0.093 | 2.031 | 0.170 |
| *Growth condition* | 1 | 1.296 | 1.296 | 28.258 | 0.000 |
| *Interaction* | 1 | 0.030 | 0.030 | 0.649 | 0.430 |
| *Residual* | 20 | 0.917 | 0.046 |  |  |
| OIR |  |  |  |  |  |
| *Maintenance condition* | 1 | 0.011 | 0.011 | 2.046 | 0.168 |
| *Growth condition* | 1 | 0.151 | 0.151 | 28.300 | 0.000 |
| *Interaction* | 1 | 0.003 | 0.003 | 0.648 | 0.430 |
| *Residual* | 20 | 0.107 | 0.005 |  |  |
| AE |  |  |  |  |  |
| *Maintenance condition* | 1 | 0.012 | 0.012 | 5.918 | 0.024 |
| *Growth condition* | 1 | 0.012 | 0.012 | 5.659 | 0.027 |
| *Interaction* | 1 | 0.001 | 0.001 | 0.384 | 0.543 |
| *Residual* | 20 | 0.041 | 0.002 |  |  |
| AR |  |  |  |  |  |
| *Maintenance condition* | 1 | 0.011 | 0.011 | 3.077 | 0.095 |
| *Growth condition* | 1 | 0.099 | 0.099 | 28.651 | 0.000 |
| *Interaction* | 1 | 0.003 | 0.003 | 0.733 | 0.402 |
| *Residual* | 20 | 0.069 | 0.003 |  |  |
| VO_2R_ |  |  |  |  |  |
| *Maintenance condition* | 1 | 0.001 | 0.001 | 4.618 | 0.044 |
| *Growth condition* | 1 | 0.000 | 0.000 | 0.608 | 0.445 |
| *Interaction* | 1 | 0.000 | 0.000 | 0.073 | 0.789 |
| *Residual* | 20 | 0.003 | 0.000 |  |  |
| VO_2S_ |  |  |  |  |  |
| *Maintenance condition* | 1 | 0.000 | 0.000 | 0.250 | 0.623 |
| *Growth condition* | 1 | 0.000 | 0.000 | 0.320 | 0.578 |
| *Interaction* | 1 | 0.000 | 0.000 | 2.050 | 0.168 |
| *Residual* | 20 | 0.003 | 0.000 |  |  |
| SFG |  |  |  |  |  |
| *Maintenance condition* | 1 | 6.177 | 6.177 | 4.708 | 0.042 |
| *Growth condition* | 1 | 32.359 | 32.359 | 24.661 | 0.000 |
| *Interaction* | 1 | 0.786 | 0.786 | 0.599 | 0.448 |
| *Residual* | 20 | 26.244 | 1.312 |  |  |

| L_H_ diet |  |  |  |  |  |
| --- | --- | --- | --- | --- | --- |
| **Source of variation** | **DF** | **SS** | **MS** | **F** | **P** |
| CR |  |  |  |  |  |
| *Maintenance condition* | 1 | 0.005 | 0.005 | 1.019 | 0.322 |
| *Growth condition* | 1 | 0.101 | 0.101 | 18.830 | 0.000 |
| *Interaction* | 1 | 0.007 | 0.007 | 1.268 | 0.270 |
| *Residual* | 26 | 0.139 | 0.005 |  |  |
| SE |  |  |  |  |  |
| *Maintenance condition* | 1 | 0.017 | 0.017 | 4.836 | 0.037 |
| *Growth condition* | 1 | 0.019 | 0.019 | 5.546 | 0.026 |
| *Interaction* | 1 | 0.001 | 0.001 | 0.300 | 0.588 |
| *Residual* | 26 | 0.091 | 0.004 |  |  |
| RP |  |  |  |  |  |
| *Maintenance condition* | 1 | 0.085 | 0.085 | 8.906 | 0.006 |
| *Growth condition* | 1 | 0.008 | 0.008 | 0.798 | 0.380 |
| *Interaction* | 1 | 0.008 | 0.008 | 0.815 | 0.375 |
| *Residual* | 26 | 0.247 | 0.010 |  |  |
| OIR |  |  |  |  |  |
| *Maintenance condition* | 1 | 0.000 | 0.000 | 0.017 | 0.898 |
| *Growth condition* | 1 | 0.085 | 0.085 | 12.275 | 0.002 |
| *Interaction* | 1 | 0.003 | 0.003 | 0.379 | 0.543 |
| *Residual* | 26 | 0.181 | 0.007 |  |  |
| AE |  |  |  |  |  |
| *Maintenance condition* | 1 | 0.028 | 0.028 | 6.737 | 0.015 |
| *Growth condition* | 1 | 0.057 | 0.057 | 13.983 | 0.001 |
| *Interaction* | 1 | 0.000 | 0.000 | 0.077 | 0.784 |
| *Residual* | 26 | 0.107 | 0.004 |  |  |
| AR |  |  |  |  |  |
| *Maintenance condition* | 1 | 0.002 | 0.002 | 0.504 | 0.484 |
| *Growth condition* | 1 | 0.061 | 0.061 | 16.801 | 0.000 |
| *Interaction* | 1 | 0.001 | 0.001 | 0.234 | 0.632 |
| *Residual* | 26 | 0.095 | 0.004 |  |  |
| VO_2R_ |  |  |  |  |  |
| *Maintenance condition* | 1 | 0.001 | 0.001 | 3.340 | 0.079 |
| *Growth condition* | 1 | 0.000 | 0.000 | 0.114 | 0.739 |
| *Interaction* | 1 | 0.000 | 0.000 | 1.452 | 0.239 |
| *Residual* | 26 | 0.008 | 0.000 |  |  |
| VO_2S_ |  |  |  |  |  |
| *Maintenance condition* | 1 | 0.000 | 0.000 | 0.975 | 0.333 |
| *Growth condition* | 1 | 0.000 | 0.000 | 0.095 | 0.760 |
| *Interaction* | 1 | 0.001 | 0.001 | 5.708 | 0.024 |
| *Residual* | 26 | 0.003 | 0.000 |  |  |
| SFG |  |  |  |  |  |
| *Maintenance condition* | 1 | 1.996 | 1.996 | 1.276 | 0.269 |
| *Growth condition* | 1 | 20.498 | 20.498 | 13.108 | 0.001 |
| *Interaction* | 1 | 0.002 | 0.002 | 0.001 | 0.970 |
| *Residual* | 26 | 40.659 | 1.564 |  |  |
